# Processing speed mediates the association between physical activity and executive functioning in elderly adults

**DOI:** 10.3389/fpsyg.2022.958535

**Published:** 2022-08-26

**Authors:** Anabela Silva-Fernandes, Sara Cruz, Célia Sofia Moreira, Diana R. Pereira, Sónia S. Sousa, Adriana Sampaio, Joana Carvalho

**Affiliations:** ^1^Psychological Neuroscience Laboratory, School of Psychology, Psychology Research Center (CIPsi), University of Minho, Braga, Portugal; ^2^The Psychology for Positive Development Research Center (CIPD), Lusíada University, Porto, Portugal; ^3^Department of Mathematics and Centre of Mathematics, University of Porto (FCUP & CMUP), Porto, Portugal; ^4^Faculty of Sport, Research Center in Physical Activity, Health and Leisure (CIAFEL), University of Porto, Porto, Portugal

**Keywords:** aging, MVPA, processing speed, executive functions, physical activity

## Abstract

Advanced aging is associated with cognitive decline. To decrease the healthcare system and socio-economic burdens as well as to promote better quality of life, is important to uncover the factors that may be related to the delay of cognitive impairments in older adults. This study investigated the relationship between physical activity levels, sedentary behavior and cardiorespiratory fitness with cognitive functioning in healthy older adults. Furthermore, it examined the mediating role of processing speed on the association between physical activity and executive functions and long-term memory. Thirty-two individuals aged between 63 and 77 years (*M* = 68.16, *SD* = 3.73) underwent measurements of maximal oxygen uptake (VO_2_peak), 1-week of PA accelerometer measurement and a comprehensive cognitive assessment. Significant associations were observed between MVPA and cognitive processing speed. Equally, a significant positive indirect effect of MVPA on executive functioning and long-term memory was mediated by processing speed. Also, MVPA levels differentiated cognitive functioning in older adults – the physical active group outperformed the physical inactive group in processing speed, executive functions, and language abilities. Our results contribute to the literature on the MVPA levels as an important tool to promote healthier cognitive aging.

## Introduction

Due to societal development (e.g., improved healthcare system) and increased longevity, the number of older adults has increased dramatically. By 2030, is expected that the world population aged 65 or over will be nearly 1 billion, reaching approximately 2 billion by 2050 (United Nations, 2019). Advanced aging encompasses diverse types of loss of function, such as physical (e.g., cardiovascular disease) or cognitive (e.g., dementia) (Niccoli and Partridge, 2012), leading to increased dependence, morbidity, and mortality (Panza et al., 2018). Indeed, age-related cognitive decline (cognitive aging) and dementia-related problems, which are associated with age-related functional loss, are a major health and socio-economic issue confronted by an increasing aging population. It is thus important to further uncover factors that are related to the delay of cognitive aging, decreasing burdens on families and healthcare systems, contributing to improvements in quality of life.

Aging is associated with changes in a multitude of cognitive tasks that require a variety of perceptual and cognitive processes. Several cognitive changes have been documented such as processing speed, memory, language, visuospatial function, and executive functions (EF) (Harada et al., 2013). Importantly, evidence suggests that older individuals, during normal aging process, experience a more significant decline in processing speed (Ball et al., 2007). Processing speed refers to the speed in which information can be perceived, understood, and responded to, which is considered part of attentional abilities (Silva and Lee, 2021). A theoretical approach had proposed that a “major factor contributing to age-related differences in memory and other aspects of cognitive function” (p. 203) is related to the ability in the speed that many cognitive tasks are executed (Salthouse, 1995, 1996). Indeed, evidence shows that age-related cognitive decline is particularly marked in processing speed abilities, which significantly decreases over time, being steeper at older ages (Shimada et al., 2018; Zaninotto et al., 2018). In turn, altered processing speed affects the loss of functioning of other cognitive domains (e.g., working memory) (Liebel et al., 2017), consequently increasing the risk for depression (Muhammad and Meher, 2021) and impairment functioning in daily-life activities (Edwards et al., 2005).

Despite this consistent evidence, cognitive aging is also a highly heterogeneous process with multiple factors contributing for inter-individual differences (Mungas et al., 2010). Among these factors, physical activity (PA) and exercise can affect brain plasticity, influencing cognition and wellbeing. Several prospective epidemiologic (Laurin et al., 2001; Yaffe et al., 2001; Barnes et al., 2003; Lytle et al., 2004; Van Gelder et al., 2004; Weuve et al., 2004), cross-sectional (Van Boxtel et al., 1997; Hillman et al., 2006) and experimental (Kramer et al., 1999; Fabre et al., 2002) studies have referred to physical activity (PA) and exercise as significant protective factors of age-related cognitive decline. The World Health Organization (WHO) released on 2020 updated guidelines on PA and sedentary behavior (Bull et al., 2020). For adults, the recommendation is 150–300 min of moderate-intensity, or 75–150 min of vigorous-intensity PA, or some equivalent combination of moderate-intensity and vigorous-intensity aerobic PA, per week. Moreover, the US Department of Health and Human Services was a pioneer in developing PA guidelines and for the first time in their 2018 edition of PA guidelines for Americans, acknowledged that there is moderate-to-strong evidence that PA can improve cognitive function in youth and adults (Erickson et al., 2019). High levels of PA and cardiorespiratory fitness (CRF) seem to have a protective effect on brain function and are associated with later onset or lower degree of age-related cognitive decline (Kramer et al., 1999; Hillman et al., 2008). For instance, higher PA levels significantly predicted increased cognitive performance in elderly, specifically on tasks involving working memory and processing speed (Bherer et al., 2013). Even unstructured PA (i.e., daily activities) seems to have a protective effect on age-related cognitive decline, with high physically active seniors presenting higher scores on full scale intelligence quotient, processing speed, matrix reasoning, digit-symbol coding and picture arrangement subtests of the Wechsler Adult Intelligence Scale (WAIS) than less active peers (Sanchez-Lopez et al., 2018). Based upon these studies, clearly engaging in physical activity may lead to cognitive benefits, which is one of the vital components necessary for successful aging.

However, despite the extensive literature showing the benefits of PA on cognitive aging, most of the studies examining the impact of PA on cognitive function have relied upon subjective assessments of PA levels (i.e., self-reports) which, in contrast to quantitative measures, namely accelerometry, fail to provide an objective and accurate estimation of non-exercise lifestyle activities such as moderate-to-vigorous PA (MVPA), light PA (LPA) and sedentary behavior (SB) (Freedson et al., 1998). On the other hand, although PA and CRF are related, they measure distinct outcomes of a physically active lifestyle. PA dose-parameters, namely intensity, may determine the magnitude of effects on cognition (Bherer et al., 2013), although the relationship between PA dose-parameters and cognitive functions is not fully understood so far. One step toward clarifying this relationship is to have more evidence that directly contrast multiple features of a physically active lifestyle and physical fitness (e.g., intensity of daily PA, sedentary behavior, CRF) thus their individual contributions to cognitive functioning can be determined.

This study objectives were threefold. First, to investigate the relationship between PA levels – (light, moderate and vigorous PA), sedentary time and CRF – and age-sensitive cognitive abilities – processing speed, language, executive functions (working memory and mental flexibility), and short and long-term memory. Afterwards, to examine the potential role of processing speed (because the loss of this ability is greatly affected by age impacting the functioning of other cognitive domains) (Salthouse, 1996, 2019) on the relationship between physical activity and executive functions and long-term memory. Lastly, to compare physical active and physical inactive groups, according to the levels of MVPA recommended by WHO regarding cognitive functioning. We hypothesized (i) a positive association between MVPA levels and higher scores on cognitive functioning measures, particularly with processing speed and executive functions; (ii) a positive indirect mediation role of processing speed on the relationship between PA and executive functions and long-term memory; and (iii) the active group to outperform the inactive group.

## Materials and methods

### Participants

Forty-seven independent community-dwelling individuals were invited to participate in this study. All participants were screened to collect the information about the inclusion and exclusion criteria. Exclusion criteria included: cognitive impairment, assessed using the Montreal Cognitive Assessment (MoCA) (Freitas et al., 2011), history of heart procedures (e.g., stents, pacemaker), neurodegenerative disorders, head injury or prior history or presence of neurological or psychiatric disorders. Years of education and socioeconomic status were also obtained, as well as information regarding frequency of PA during the previous six months. Five participants were excluded due to the presence of chronic depression disorder, four for history of heart procedures, two for having suffered a stroke, one participant was excluded due to cognitive impairment and three for having depression or anxiety symptoms. Therefore, 32 participants aged between 63 and 77 years old (*M* = 68.16; *SD* = 3.73), constitute the final sample enrolled in this study. Additionally, two participants were excluded in the group-comparative analysis based on WHO recommendations for PA due to missing values in accelerometer assessment and four participants have missing for VO_2_peak.

All participants lived in their own homes and were able to perform activities of daily life independently. The nature, benefits, and risks of the study were explained to the volunteers and written informed consent was obtained, consistent with the Declaration of Helsinki. The Ethics Subcommission of Life and Health Sciences (SECVS 120/2016) approved all methods and procedures implemented in our investigation.

### Physical fitness and PA measurement

#### Aerobic fitness

Aerobic fitness assessment was conducted by the Modified Bruce protocol on a treadmill (Quasar, h/p/cosmos, Germany) with a standard open-circuit spirometer technique (Oxycon Pro Metabolic Cart, Jaeger, Carefusion, Germany) to assess VO_2_max. The first stage of the Modified Bruce Test is performed at a 2.7 Km/h and 0% grade and the second stage corresponds to the first stage of the Standard Bruce Test protocol (Bruce et al., 1949a,b).

Oxygen uptake (VO_2_), carbon dioxide production (VCO_2_), heart rate (1/min) and respiratory exchange ratio (RER; a ratio of metabolic gas exchange calculated by VCO_2_ divided by VO_2_) were collected. The maximal effort of the participant was defined when two of the following criteria were met: (i) a plateau in VO_2_ with further increases in workload; (ii) a RER ≥ 1.0, and (iii) maximal heart rate ≥ 85% of the age-predicted maximal heart rate (i.e., 220-age). Most of the participants did not achieve full exertion since objective outcomes previously mentioned or subjective measures (Borg Scale > 8) were not met. Consequently, aerobic capacity was defined as VO_2_peak in milliliter per kilogram body weight per minute (ml/kg/min) rather than VO_2_max. VO_2_peak was reported as the 30-s mean VO_2_ of the highest complete performance level achieved by the participants.

#### PA levels

For the evaluation of habitual PA, participants were instructed to wear the GT3X Actigraph accelerometer (ActiGraph; Pensacola, Florida) on the right hip (close to the iliac crest) for 7 consecutive days, during all waking hours except for showering or participating in water-based activities. For the purposes of this analysis, a valid day of data consisted of at least 8 h of valid wear-time, where non-valid wear time were defined as 60 min of consecutive zeros. Only data from individuals with a minimum of 4 valid days (3 weekdays and 1 weekend day) were included for processing with the ActiLife v6.0 software. One-min register provided 60 samples of PA per hour. The cut-off points described by Troiano (Troiano et al., 2008) were used, such as sedentary activity defined as 0–99 counts/min, light physical activity (LPA) defined as 100–2019 counts/min and moderate-to-vigorous physical activity (MVPA) defined as > 2020 counts/min.

According to the minimum recommendations of MVPA activity for adults (30 min/day 5 times per week) defined by the WHO (2010) to improve cardiorespiratory and muscular fitness, bone and functional health, reduce the risk of noncommunicable diseases, depression and cognitive decline, we classified our participants in two groups: 1) Physical Inactive (PIn) (MVPA <30 min/day) and 2) Physical Active (PAc) (MVPA≥30 min/day).

### Cognitive performance assessment

Participants' general cognitive functioning was assessed with the MoCA test (Freitas et al., 2010). MoCA is widely used as a screening test for cognitive function in aging. It provides great sensitivity in identifying cognitive decline as it assesses eight cognitive domains: executive functions, visuospatial abilities, short-term memory, language, attention, concentration and working memory. Additionally, considers temporal and spatial orientation. Afterwards, a cognitive assessment battery was administered and included the following tests:

i) Trail making test – A and B (TMT-A, TMT-B) (Cavaco et al., 2013b), to assess processing speed and mental flexibility, respectively;

ii) Symbols Search subtest from Wechsler Adult Intelligence Scale (3^rd^ edition) – WAIS-III (Wechsler, 1997), to assess attention and processing speed competences;

iii) Phonemic and semantic fluency test (Cavaco et al., 2013a), to measure non-motor processing speed, language production and executive functions;

iv) Vocabulary test (WAIS-III) (Wechsler, 1997);

v) Digit span subtest (WAIS-III, 1997) (Wechsler, 1997), to assess attention, short-term memory (direct span direct order) and working memory (digit span inverse order);

vi) Visual reproduction I and II, Logical memory I and II and the Verbal paired associates I and II subtests from the Wechsler Memory Scale (WMS-IV, 2009) (Wechsler, 2009), to assess short and long-term memory abilities, respectively.

Afterwards, five composites were computed to represent cognitive domains: (i) language (phonemic and semantic fluency test and vocabulary subtest), (ii) executive functions (TMT-B^inv^ and digits span backward), (iii) processing speed (symbols, TMT-A^inv^), (iv) short-term memory (STM) (visual reproduction I, logical memory I, verbal paired associates I), and v) long-term memory (LTM) (visual reproduction II, logical memory II, verbal paired associates II). The composites were created by combining the tests that assessed the same cognitive domain, as it has psychometric advantages over individual tests [for example see (Gibbons et al., 2012)].

For each composite, raw scores of the tests were put into the same range (percentage), and then averaged and standardized. The time spent for TMT-A and -B were inverted immediately after the first step of the parceling procedure.

### Depression and anxiety assessment

Depression and anxiety symptoms were assessed using the Geriatric Depression Scale (GDS) and the Geriatric Anxiety Inventory (GAI) (Ribeiro et al., 2011; Hughes et al., 2013), respectively.

### Procedure

Independent community-dwelling elderly adults were contacted before starting the first or second year of “Mais vividos, mais ativos,” a senior physical exercise program at Faculty of Sport of the University of Porto (FADEUP). Others were invited by personal invitation in the community. All participants were invited to fill in a checklist regarding the inclusion/exclusion criteria (first screenining) in order to determine their participation in this study. Then, those who met the criteria were invited for a session in which the responsible researcher explained the study objetives and procedures. Those who manifested interest in participating signed the informed consent. Afterwards, individual cognitive and physical assessment sessions were scheduled, which occurred in a quiet room. Then, seniors were instructed to use accelerometer, as previously described above, during 1 week.

### Statistical data analysis

#### Power analysis and sample size

When planning this study, the calculation of the minimum number of participants needed was performed. For this purpose, the number of individuals enrolled would have to provide enough power to detect, at least, large effect sizes.

The minimum sample size was estimated using the R package WebPower (Zhang and Mai, 2018) and, as data would be analyzed through linear models, a regression effect power analysis was conducted – selecting 80% of power, 5% of type I error probability, and effect size f^2^ = 0.35 (Cohen, 1988). This calculation indicated that a minimum of 25 subjects were needed to proceed with this study.

#### Data analysis

Statistical analyses were performed using R statistical environment (R Studio, version 3.6.2, R Core Team, 2019). An alpha level of 0.05 was used for significance.

Mean and standard deviation were computed for all variables that were used in the screening stage, displayed by sex. Pearson correlations were calculated to explore the relationship between sedentary behavior, PA levels (LPA and MVPA) and aerobic fitness (VO_2_peak) with cognitive performance.

**Figure 1:**
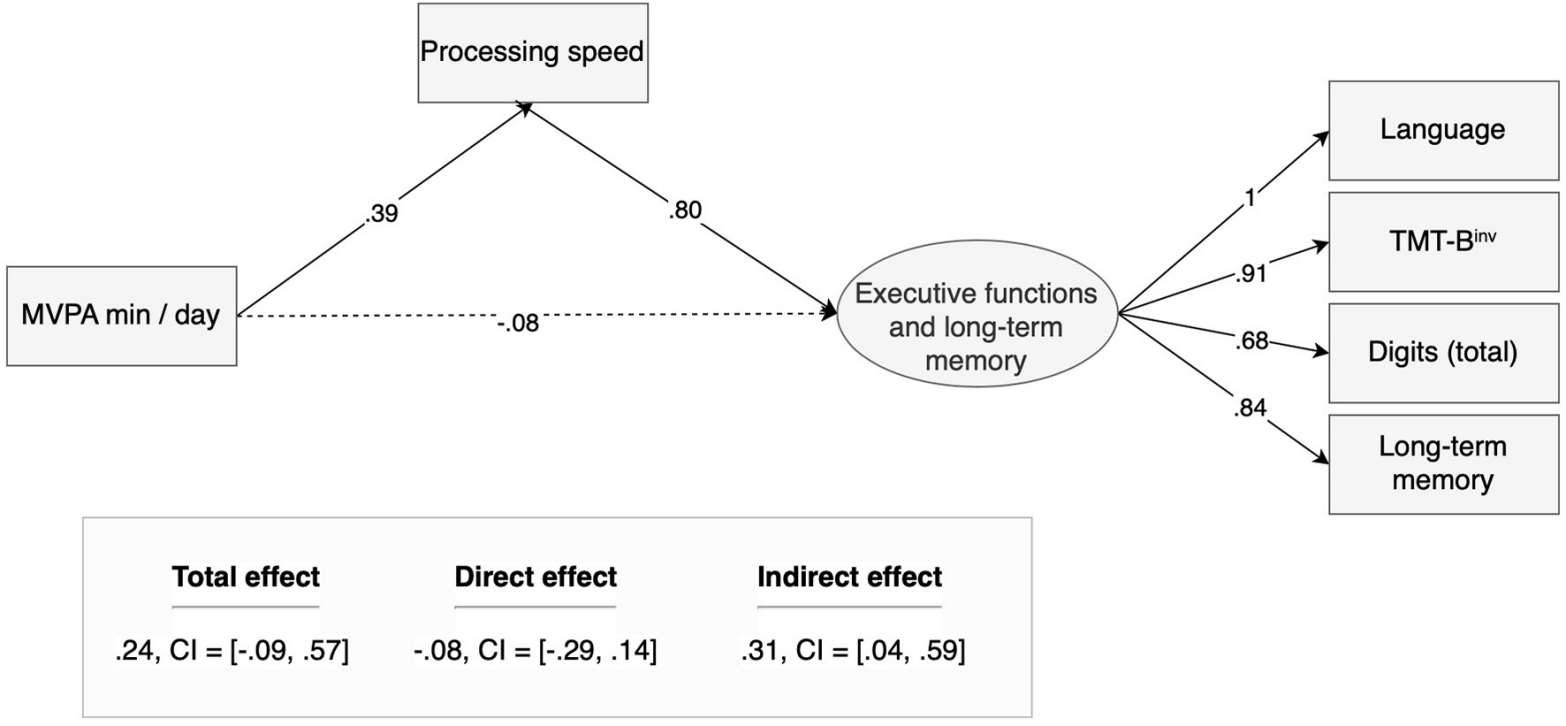
The mediation model of the effect of MVPA on executive functions and long-term memory through processing speed.

A mediation model was performed to evaluate the effect of MVPA on executive functions and long-term memory through processing speed. In the mediation model, bootstrap estimates of standard errors were computed (Shrout and Bolger, 2002; Hoyle, 2012). From the statistical point of view, we created a latent variable “EF and Long-term memory” using the following four indicators: “TMT-B^inv^,” “total digits,” “language,” “long-term memory.” A confirmatory factor analysis showed that this latent construct had a good reliability, with all factor loadings being statistically significant (*p* < 0.001) and higher than 0.69. Moreover, the Cronbach's alpha was 0.86 and the average variance extracted (AVE) was 0.61, meaning that the latent variable had very good internal consistency and validity. In the mediation model, all observed variables were standardized and centered. Moreover, the residual variances of the latent variables were fixed to 1. Figure 1 outlines and summarizes the mediator effect, which was estimated using a bootstrap procedure (Shrout and Bolger, 2002; Hoyle, 2012). The estimate is 0.37 with 95% bootstrap CI = (0.07,0.75).

Considering the MVPA levels recommended in the WHO guidelines, we further compared the PAc and PIn groups regarding cognitive functioning. Differences between groups (female vs. male, PAc vs. PIn) were assessed through regression modeling. This statistical technique has advantage over traditional statistical tests because it allows using an adequate family of distributions for each outcome, as well as obtaining an estimate for each difference. For positive discrete data, the Conway-Maxwell-Poisson distribution was used. Such models were performed using the R package “glmmTMB” (Brooks et al., 2017) being interpreted as log-linear models. For gender, we used logistic regression. All the other continuous variables were assessed through linear regression modeling. Outliers were assessed for all variables. Whenever outliers were identified for a given outcome, the corresponding model was also analyzed without those extreme observations and the results of both models were compared. In all cases, the differences did not produce changes in the significance of the results and so, for simplicity, we only present the output for the complete data.

## Results

Table 1 depicts the descriptive statistics for social-demographic characteristics, anthropometric measures (BMI) and general cognitive functioning (MoCA test), anxiety (GAI) and depressive symptomatology (GDS). No sex differences were observed regarding social-demographic, anthropometric, cognitive, and affective measures. Of note, cognitive performance was within the normative range of the age-matched population.

**Table 1:** Sex differences, comparing men (reference level) to women.

	**Total** **(*N* = 32)** **M (SD)**	**Male** **(*n* = 13)** **M (SD)**	**Female** **(*n* = 19)** **M (SD)**	**Regression**	**Diff**	**SE**	***p*-value**
Age (years)	68.16 (3.73)	66.92 (3.50)	69.00 (3.73)	CMP	0.03	0.02	0.098
Education (years)	9.84 (4.31)	8.33 (3.55)	10.79 (4.57)	CMP	0.26	0.16	0.101
BMI (Kg/m^2^)	28.84 (3.55)	29.96 (2.91)	28.07 (3.82)	linear	−1.89	1.25	0.141
MoCA	23.72 (3.31)	24.54 (3.53)	23.16 (3.13)	CMP	−0.06	0.05	0.230
GDS	2.77 (2.50)	3.00 (3.07)	2.61 (2.12)	CMP	−0.14	0.31	0.658
GAI	2.47 (2.81)	2.17 (3.10)	2.67 (2.68)	CMP	0.21	0.44	0.634

*M, Mean; SD, Standard deviation; BMI, Body Mass Index; MoCA, Montreal Cognitive Assessment; GDS, Geriatric Depression Scale; GAI, Geriatric Anxiety Inventory; Diff, Estimate between-groups difference; SE, Standard error; CMP, Conway-Maxwell-Poisson distribution*.

### Correlation analysis

Table 2 presents Pearson correlation values between aerobic fitness (VO_2_peak), PA (MVPA, LPA) and sedentary levels, cognitive scores, age, and years of education.

**Table 2:** Correlation analysis between physical activity measures and cognitive performance.

	**1**	**2**	**3**	**4**	**5**	**6**	**7**	**8**	**9**	**10**	**11**
1. Age	1										
2. Education	−0.01	1									
3. VO_2_peak	−0.33	−0.02	1								
4. MVPA	−0.25	0.36	0.33	1							
5. LPA	−0.32	−0.05	0.09	0.09	1						
6. Sedentary	0.15	−0.05	−0.02	−0.36*	−0.59*	1					
7. Language	−0.14	0.38*	0.00	0.22	−0.13	0.21	1				
8. Executive functions	−0.32	0.45*	0.16	0.31	−0.03	−0.03	0.77*	1			
9. Processing Speed	−0.22	0.41*	0.10	0.40*	−0.03	0.00	0.76*	0.68*	1		
10. Short-term memory	−34	0.18	−0.04	0.02	−0.16	0.08	0.59*	0.63*	0.58*	1	
11. Long-term memory	−0.32	0.23	0.08	0.19	−0.19	0.09	0.69*	0.62*	0.65*	0.86*	1

MVPA, Moderate-to-vigorous physical activity; LPA, Light physical activity; phon, phonemic fluency test; sem, semantic fluency test; voc, vocabulary; TMT-B^inv^, Trail-making test B inverse; TMT-A^inv^, Trail-making test A inverse. Cognitive domains composites: Language (phon, sem, voc); Executive functions (TMT-B^inv^, inverse digits); Processing Speed (Symbols, TMT-A^inv^); Short-term memory (Visual reproduction I, Logical memory I, Verbal paired associates I); Long-term memory (Visual reproduction II, Logical memory II, Verbal paired associates II). Correlation values using two decimal places. *p < 0.05.

Years of education was positively associated with language (*p* = 0.034), executive functions (*p* = 0.010) and processing speed (*p* = 0.021) composites. MVPA level was positively associated with processing speed composite (*p* = 0.029).

No further associations were found between VO_2_peak, LPA, sedentary time, and cognitive measures.

### From correlations and regression models to mediations

The main objective of this study was to investigate the relation between PA metrics (LPA and MVPA), sedentary behavior and cardiorespiratory fitness with cognitive functioning in healthy older adults.

First, we analyzed the correlation coefficients (Table 2). As measures of linear association, they allowed us to draw a preliminary idea about the relationships of interest. All cognitive measures are strongly correlated. Of these, only processing speed was significantly associated with MVPA. Therefore, we used statistical methodology to assess the relation of MVPA and cognitive performance (Pearl and Mackenzie, 2018). Before assuming the relationship, it was necessary to ensure that it was not being confounded by common causes of both the dependent and the independent variable (Pearl and Mackenzie, 2018). Note that these common causes needed to be correlated with both variables. An inspection of Table 2 confirmed that none of the variables in this study could be a traditional confounding variable of this relationship (although there may exist other confounding variables not considered here). Therefore, we assumed the relationship between MVPA and processing speed. In fact, previous literature demonstrated significant associations between MVPA and processing speed in Multiple Sclerosis patients (Motl et al., 2022) as well as in healthy subjects (Erickson et al., 2019).

Second, we used standardized variables and linear regression analysis to further investigate this relationship. The linear model describing processing speed as a function of MVPA, exhibited a significant estimate β_MVPA_ = 0.41 (*p* = 0.029). Following recent recommendations about “good and bad controls” (Cinelli et al., 2022), we investigated possible causes of processing speed, since their inclusion in the model could improve the effect obtained from MVPA. Considering Table 2, we added “education” to the previous linear model and observed that the estimate slightly increased (β_MVPA_ = 0.49, *p* = 0.009; β_education_ = 0.07, *p* = 0.082), a fact that strengthened the hypothesized relationship between MVPA and processing speed. Nonetheless, we also confirmed that adding other covariates – age, sex, and VO2peak – to the initial linear model did not produce significant changes in this relationship (Supplementary Table 1). It is worth noting that theoretically, the other cognitive measures cannot be considered causes of processing speed (Salthouse, 1996), and thus these variables should not be included as controls in the relationship between MVPA and processing speed (Cinelli et al., 2022).

Regarding the other cognitive measures, we confirmed that the linear models describing them as functions of MVPA did not yield significant regression coefficients (language: β_MVPA_ = 0.23; executive functions: β_MVPA_ = 0.32; short-term memory: β_MVPA_ = 0.02; long-term memory: β_MVPA_ = 0.19) (see Supplementary Table 1). Moreover, when adding covariates, we observed that only the relationship between MVPA and language became significantly stronger after controlling for education (β_MVPA_ = 0.36, *p* = 0.046; β_education_ = 0.07, *p* = 0.087).

Finally, a third analysis was conducted. In fact, the evidence of the relationship between MVPA and processing speed, and the high correlations between all cognitive measures, lead us to suspect that processing speed could be transmitting part of the effect received from MVPA to the remaining cognitive domains. This hypothesis was further investigated using mediation analysis. Mediation analysis has been the subject of several discussions and developments in the last decades. In this paper, we follow the most recent recommendations and used the Structural Equations Modeling (SEM) approach (MacKinnon, 2008). Thus, to claim the mediation of a variable *M* in a *X*→*Y* relationship, we consider the following two classical linear regression models:

*M* = *i*_1_ + *aX* + *e*_1_,

*Y* = *i*_2_ + *bM* + *c' X* + *e*_2_,

where *i*_1_ and *i*_2_ are the intercepts, *a, b*, and *c* are the regression coefficients, and *e*_1_ and *e*_2_ are residuals; and require the quantities *a, b*, and the product *a*×*b* to be significantly nonzero. This product is known in the literature as the indirect effect or the amount of mediation. The direct effect is the coefficient *c'* relating the causal variable to the outcome adjusted for the mediator.

### The mediation model

The potential mediating role of processing speed in the relationship between MVPA with executive functions and long-term memory was examined.

Using mediation analysis, we confirmed the existence of three individual mediated effects. More precisely, only short-term memory did not show a significant indirect effect from MVPA mediated by processing speed. For the sake of simplicity, these individual cognitive abilities were gathered in a structural equation mediation model (Figure 1). Confirmatory factor analysis showed very good reliability of this latent variable: all factor loadings were significant and higher than 0.65, construct reliability CR = 0.86, and average variance extracted AVE = 0.61 (Fornell and Larcker, 1981). Moreover, although the existence of the mediation effect is independent of the goodness of fit indices, very good measures indicated that this hypothesized model fitted adequately the observed data (Hu and Bentler, 1999): Chi-square χ^2^ = 8.773, df = 8, *p* = 0.362, CFI = 0.991, TLI = 0.984, RMSEA = 0.057, SRMR=0.043. Considering previous results on linear modeling, we further considered controlling for participants' education. However, this control introduced only small perturbations in the parameter estimates and worsened the fit indices, confirming the superiority of the model outlined in Figure 1.

Importantly, this mediation allows us to better understand the relationship between MVPA and executive functions and long-term memory. More specifically, it “unveils” significant indirect effects of MVPA on language, executive functions, and long-term memory, which are impossible to detect in a more elementary correlational analysis, or even using regression analysis. A significant positive indirect effect of MVPA on EF and LTM mediated by processing speed was observed [95% bootstrap confidence interval = (0.04, 0.59), see Figure 1]. After controlling for this mediator effect, no direct effect was observed between MVPA and EF and LTM.

Interestingly, in this case, the mediator effect is also called a suppressor effect. Suppression occurs when the direct effect and the indirect effect have opposite signs, and it “can explain why a theoretically interesting relation is not strong” (Shrout and Bolger, 2002). Therefore, this model shows two opposite effects from MVPA on executive functions and long-term memory: a positive effect mediated by processing speed, and a negative direct effect obtained after controlling for the mediator. It is worth noting that this negative direct effect is weak (nonsignificant) but is responsible for the nonsignificant total effect. This means that participants with higher levels of MVPA showed, in general, higher levels of processing speed, which in turn were associated with higher levels of executive functions and long-term memory. However, although weak, after controlling for this mediator it was also possible to observe a negative direct effect. In general, this result shows the important contribution of MVPA in improving processing speed, as well as the important role of this cognitive ability in improving other cognitive skills.

### Group-comparative analysis based on WHO recommendations for PA

We further explored the neurocognitive profile in two groups classified accordingly with this MVPA threshold (PAc vs. PIn). No group differences were observed for age, years of education and VO_2_peak, as these were potential confounding factors for cognitive outcomes (Table 3). As expected, MVPA levels were significantly different between groups with no differences being observed in LPA and sedentary time (Table 3).

**Table 3:** Between-groups differences, comparing the PIn group (reference level) to the PAc group.

		**PIn (*n* = 14)** **M (SD)**	**PAc** **(*n* = 16)** **M (SD)**	**Diff**	**SE**	***p*-value**
Sex	F	8	10	0.22	0.75	0.765
	M	6	6			
Age		69.36 (4.01)	67.19 (3.10)	−2.17	1.30	0.106
Education		8.71 (3.79)	11.20 (4.55)	2.49	1.56	0.123
MVPA		16.25 (6.96)	52.84 (17.05)	1.61	0.21	<0.001***
LPA		331.08 (78.78)	338.74 (94.28)	0.09	0.37	0.812
Sedentary		421.57 (94.62)	383.83 (80.19)	−0.43	0.36	0.247
VO_2_peak		23.66 (4.73)	25.35 (4.61)	0.37	0.40	0.360
HR		140.42 (13.49)	139.73 (9.96)	−0.06	0.39	0.881
RER		1.09 (0.11)	1.04 (0.09)	−0.50	0.39	0.209
Language		−0.45 (0.76)	0.38 (1.08)	0.83	0.35	0.023*
Executive functions		−0.49 (0.93)	0.36 (0.96)	0.85	0.35	0.021*
Processing speed		−0.51 (0.87)	0.44 (0.96)	0.95	0.34	0.009**
Short-term memory		−0.08 (1.12)	0.14 (0.93)	0.27	0.37	0.464
Long-term memory		−0.24 (1.05)	0.27 (0.94)	0.55	0.36	0.132

*p < 0. 05; **p < 0.01; ***p < 0.001.

*PIn, Physical Inactive group; Pac, Physical Active group; M, Mean; SD, Standard deviation; MVPA, Moderate-to-vigorous physical activity; VO2peack, Peak oxygen uptake; HR, Heart rate; RER, Respiratory Exchange Ratio; TMT-B, Trail making test B; TMT-A, Trail making test; Diff, Between-groups difference estimate; SE, Standard error. For gender assessment, a logistic model was computed; all the other cases were assessed through robust regression modeling*.

Globally, the PAc group performed significantly better than the PIn group in language, executive functions and processing speed tests (Table 3).

## Discussion

Aging is accompanied by declines in a range of cognitive abilities (Hedden and Gabrieli, 2004). In this sense, it is of great interest to study the factors that may modulate and preserve cognitive health. With the present work, we aimed at studying the role of physical activity intensity, sedentary behavior, and cardiorespiratory fitness on cognitive functions. Specifically, we investigated the association of LPA and MVPA levels, sedentary time and VO_2_peak with specific cognitive functions in a sample of healthy older adults, using a comprehensive range of tests addressing age-sensitive cognitive abilities.

Significant associations were observed between MVPA levels with processing speed. These findings seem to be in line with previous evidence demonstrating an association between PA and cognitive performance (Barnes et al., 2003; Sanchez-Lopez et al., 2018). Our findings seem to be in line with other studies showing that increased PA levels may be related with better executive functioning, promoting attention, processing speed or working memory abilities (Bherer et al., 2013). Also, previous cross-sectional and longitudinal studies have linked increased levels of PA and preserved cognitive performance, particularly executive functions (Colcombe and Kramer, 2003; Brown et al., 2010; Netz et al., 2011; Prakash et al., 2011; Guiney and Machado, 2013).

Regarding the relationship between cognitive performance and cardiorespiratory fitness we did not find significant associations, which has been previously evidenced (Kramer and Colcombe, 2018). These may be due to some limitations of VO_2_peak measurement in elderly population. It has been showed that older adults might not be capable of producing maximal effort in CRF testing (Church et al., 2008) due to the level of motivation, perceived exhaustion and/or muscular weakness/fatigue (Evans et al., 2015), which can result in underestimation of aerobic capacity. This limitation might be concealing the relationship with cognition-related outcomes (Dougherty et al., 2017). It is also important to remark that our elderly sample is enriched with people that have a high active life routine (54% achieve MVPA 30 min/day or above) in comparison with other studies with lower percentage of people achieving the 30 min/day (10%), which might influence our correlations analysis between PA, CRF and cognitive performance. In addition, due to the exclusion and inclusion criteria needed for the study, our sample of elderly adults has lower disease burden that may not reflect the reality of elderly population. In this manner, our findings on the mediating role of processing speed between MVPA and EF are expected to be more evident in a more heterogeneous sample. Although we found positive correlations between speed processing with MVPA we think that more studies are needed with larger sample size.

We further explored the relation between LPA and sedentary time with cognitive function. However no significant results were found. This might partially be explained by the fact that the higher MVPA levels observed in this sample could cover the putative effect of LPA on cognition.

Then, we pursued the relation between MVPA levels and cognitive functions to better understand the direct and indirect effect on different cognitive domains. In fact, our results demonstrated that MVPA have an indirect effect on executive functions and long-term memory mediated by processing speed. No direct effects were observed of MVPA on these cognitive abilities. The direct effect of MVPA on processing speed is in line with the literature as well as with systematic reviews demonstrating that processing speed is one of the most stable and consistent cognitive functions related to physical exercise (Gomes-Osman et al., 2018; Falck et al., 2019). According to our results, processing speed may be enhanced by physical activity in healthy elder population (Nouchi et al., 2014; Frederiksen et al., 2015), which in turn contributes to the improvement of executive functions (Bherer et al., 2013; Salthouse, 2019). Particularly, these findings highlight processing speed as a possible target component that may function as a lever to improve executive functioning in healthy elderly adults (Guiney and Machado, 2013). This study adds to a large body of research indicating that physical activity could contribute to delaying the negative impact of age on cognitive performance (Xiong et al., 2021).

In accordance with the pattern of results observed between MVPA and cognitive functioning and given the WHO recommendations for the minimum MVPA levels of activity practice for better healthy status, which is particularly important to prevent cognitive decline, an additional aim of our study was to explore the MVPA effect on executive functions (verbal fluency, processing speed, working memory and mental flexibility) and LTM. In this manner, we have split the elderly adults according to the cut off recommended by the WHO guidelines for PA (30 min/day) and compared their cognitive performance (physically inactive – Pin – and active – Pac).

Our results showed that the Pac older adults significantly outperformed the Pin group, in specific measures of processing speed, language and executive functioning. Our findings seem to be in line with other supporting evidence suggesting that the Pac elderly, that is, those achieving or exceeding 30 min/day of MVPA levels, seem to have increased cognitive performance. In a cross-sectional study (Kerr et al., 2013), it has been showed that 30 min per day of MVPA was associated with approximately 20% greater processing speed demonstrated in executive control tasks. This result highlights the importance of the relationship between PA intensity and cognitive functioning. Similarly, in another study, increased levels of PA were associated with greater processing speed and executive functioning abilities, but not with memory performance (assessed using the Ray Verbal Memory Test) (Desjardins-Crépeau et al., 2014). Overall, and in accordance with the existent literature, it seems that even moderate PA levels might relate positively to better cognitive abilities, particularly processing speed, working memory and verbal fluency.

Cross-sectional studies (Hillman et al., 2006) have suggested that PA might be efficient in slowing the progression of the age-related cognitive decline or even enhance cognitive capacities. In the same line, longitudinal studies provide important information regarding PA impact on cognitive functioning. Specifically, a longitudinal study with more than 4,500 adults (aged 50 years or older) revealed that PA and executive function were strictly interlinked, in which changes in executive function reflect changes in PA levels (Daly et al., 2015). Other evidence, hypothesize that PA can buffer the effects of aging on cognitive decline, mainly in executive functions (Weinstein et al., 2012; Erickson et al., 2013). PA may ameliorate the impact of aging on the cognitive abilities of the elderly through a variety of complex and multifactorial physiological mechanisms. In fact, PA has been demonstrated to increase neuron survival and neuroplasticity through activation of blood circulation, bringing more oxygen, neurotrophins (BDNF), hormones (endorphins and monoamines) and nutrients to the brain (Harber and Sutton, 1984; Lin and Kuo, 2013; Sleiman et al., 2016). Additionally, it has also been shown that PA activates a cascade of cellular events that lower the number of pro-inflammatory cells and reduce pro-inflammatory cytokine production thus reducing the chronic levels of inflammation that typically arise throughout the aging brain process (De Martinis et al., 2005; Beavers et al., 2010; You et al., 2013).

The current study contributes to the extant literature by extending the processing speed hypothesis in aging to encompass that cognitive processing speed mediates the effect of MVPA to EF. Overall, our results seem to be in accordance with the extensive line of investigation demonstrating the importance of MVPA as a sensitive and accurate measure associated with increased cognitive performance in elderly adults. In addition, we observed the mediating role of processing speed in the relationship between MVPA and executive functions. Although our results are in line with other evidence, these should be analyzed with caution, mainly due to our sample size.

It is important to note that cross-sectional mediation has received some criticism mainly due to a misinterpretation of two recent articles (Maxwell and Cole, 2007; Maxwell et al., 2011), where it is shown “that cross-sectional approaches to mediation typically generate substantially biased estimates of longitudinal parameters” (Maxwell et al., 2011, p. 816). It is important to highlight the words “longitudinal parameters” in this statement. This warning applies only to longitudinal mediation, not to cross-sectional mediation. Regarding this topic, we clarify that the mediation presented in this work is cross-sectional; it is not a longitudinal mediation. This means that our model is not considering (and does not intend to infer) temporal sequences of causal effects between the included variables. Indeed, all variables of this study represent individual measures or characteristics at a specific time point. The mediation effect of processing speed is supported by the above definition of mediation effect, within the SEM framework.

This work has some limitations. Importantly, the number of participants enrolled. Future studies should replicate this work with a larger number of participants. Also, it would be interesting for future studies to address the influence of Socioeconomic Status (SES) or of life-time habits (e.g., history of physical exercise or diet) on the relationship between PA and cognitive functioning. Moreover, more studies are needed to identify an accurate threshold of the optimum intensity of PA to obtain the maximum benefits to cognitive health and, perhaps, complement the current PA guidelines (Brown et al., 2012; Zhu et al., 2015). It has been described that working memory and processing speed are early and highly affected by cognitive aging (Finkel et al., 2007). So, it is of extreme importance to understand how environmental life-style factors may be modulating cognitive processes. Future investigations should examine if a threshold amount of PA and sedentary behavior is required to maintain cognitive health in older adults.

## Data availability statement

The raw data supporting the conclusions of this article will be made available by the authors, without undue reservation.

## Ethics statement

The studies involving human participants were reviewed and approved by the Ethics Subcommittee of Life and Health Sciences, University of Minho (SECVS 120/2016). The patients/participants provided their written informed consent to participate in this study.

## Author contributions

AS-F wrote the manuscript, carried out subject recruitment and assessment, participated in the study design and data acquisition protocol, and carried out the database, and the preliminary statistical analysis. SC collaborated in subject assessment, data collection, and manuscript writing. CM performed the statistical analysis and collaborated in manuscript writing. DP collaborated in subject assessment and data collection. SS collaborated in data analysis and revision of the paper. AS and JC participated in the study design, data analysis, and writing revision. All authors read and approved the final manuscript.

## Funding

This study was conducted at the Psychology Research Center (PSI/01662), School of Psychology, University of Minho, supported by the Foundation for Science and Technology (FCT) through the Portuguese State Budget (Ref.: UIDB/PSI/01662/2020) and at Research Center in Physical Activity, Health and Leisure (FCT/ UIDB/00617/2020), University of Porto, through national funds (PIDDAC) and co-funded by FEDER through COMPETE2020 under the PT2020 Partnership Agreement POCI-01-0145-FEDER-031808). This study was also supported by Instituto Português do Desporto e Juventude (IPDJ). SC was supported by the Portuguese Foundation for Science and Technology through national funds (UID/PSI/04375/2019). CM was partially supported by CMUP, which is financed by national funds through the Portuguese Foundation for Science and Technology (FCT), under the project with reference [UIDB/00144/2020]. AS-F was supported by the Portuguese Foundation for Science and Technology and the Portuguese Ministry of Science, Technology and Higher Education, through the national funds, within the scope of the Transitory Disposition of the Decree No. 57/2016, of 29th of August, amended by Law No. 57/2017 of 19 July and previously through the fellowship grant SFRH/BPD/107732/2015. SS was supported by the project PTDC/PSI-ESP/28228/2017, funded by the Portuguese Foundation for Science and Technology (FCT) and the European Regional Development Fund (FEDER). AS was funded by Bial Foundation (#286/16), FCT (NORTE-01-0145-FEDER-032152, POCI-01-0145-FEDER-028682). JC was funded by FCT (POCI-01-0145-FEDER-031808) and by Instituto Português do Desporto e Juventude.

## Conflict of interest

The authors declare that the research was conducted in the absence of any commercial or financial relationships that could be construed as a potential conflict of interest.

## Publisher's note

All claims expressed in this article are solely those of the authors and do not necessarily represent those of their affiliated organizations, or those of the publisher, the editors and the reviewers. Any product that may be evaluated in this article, or claim that may be made by its manufacturer, is not guaranteed or endorsed by the publisher.
