# Corrigendum: Processing speed mediates the association between physical activity and executive functioning in elderly adults

**DOI:** 10.3389/fpsyg.2022.1049553

**Published:** 2022-10-06

**Authors:** Anabela Silva-Fernandes, Sara Cruz, Célia Sofia Moreira, Diana R. Pereira, Sónia S. Sousa, Adriana Sampaio, Joana Carvalho

**Affiliations:** ^1^Psychological Neuroscience Laboratory, School of Psychology, Psychology Research Center (CIPsi), University of Minho, Braga, Portugal; ^2^The Psychology for Positive Development Research Center (CIPD), Lusíada University, Porto, Portugal; ^3^Department of Mathematics and Centre of Mathematics, University of Porto (FCUP & CMUP), Porto, Portugal; ^4^Faculty of Sport, Research Center in Physical Activity, Health and Leisure (CIAFEL), University of Porto, Porto, Portugal

**Keywords:** aging, MVPA, processing speed, executive functions, physical activity

In the published article, there was an error in the **Funding** statement as published. The following statement was missing: “This study was also supported by Instituto Português do Desporto e Juventude (IPDJ).” The correct Funding statement appears below.

## Funding

This study was conducted at the Psychology Research Center (PSI/01662), School of Psychology, University of Minho, supported by the Foundation for Science and Technology (FCT) through the Portuguese State Budget (Ref.: UIDB/PSI/01662/2020) and at Research Center in Physical Activity, Health and Leisure (FCT/ UIDB/00617/2020), University of Porto, through national funds (PIDDAC) and co-funded by FEDER through COMPETE2020 under the PT2020 Partnership Agreement POCI-01-0145-FEDER-031808). This study was also supported by Instituto Português do Desporto e Juventude (IPDJ). SC was supported by the Portuguese Foundation for Science and Technology through national funds (UID/PSI/04375/2019). CM was partially supported by CMUP, which is financed by national funds through the Portuguese Foundation for Science and Technology (FCT), under the project with reference [UIDB/00144/2020]. AS-F was supported by the Portuguese Foundation for Science and Technology and the Portuguese Ministry of Science, Technology and Higher Education, through the national funds, within the scope of the Transitory Disposition of the Decree No. 57/2016, of 29th of August, amended by Law No. 57/2017 of 19 July and previously through the fellowship grant SFRH/BPD/107732/2015. SS was supported by the project PTDC/PSI-ESP/28228/2017, funded by the Portuguese Foundation for Science and Technology (FCT) and the European Regional Development Fund (FEDER). AS was funded by Bial Foundation (#286/16), FCT (NORTE-01-0145-FEDER-032152, POCI-01-0145-FEDER-028682). JC was funded by FCT (POCI-01-0145-FEDER-031808) and by Instituto Português do Desporto e Juventude.

The authors apologize for this error and state that this does not change the scientific conclusions of the article in any way. The original article has been updated.

## Publisher's note

All claims expressed in this article are solely those of the authors and do not necessarily represent those of their affiliated organizations, or those of the publisher, the editors and the reviewers. Any product that may be evaluated in this article, or claim that may be made by its manufacturer, is not guaranteed or endorsed by the publisher.

