# Supplementary material for: Processing speed mediates the association between physical activity and executive functioning in elderly adults
Source: Front Psychol. 2022 Aug 26;13:958535. doi: 10.3389/fpsyg.2022.958535 (PMC9462456; doi:10.3389/fpsyg.2022.958535)
Supplement: Supplementary file 1 [file Table_1.DOCX]

Supplementary Table 1 – Results of the linear models describing (standardized) cognitive outcomes as a function of MVPA

| Outcome | Control C | β_MVPA_ (*p*-value) | β_C_ (*p*-value) |
| --- | --- | --- | --- |
| Processing speed | -- | **.41** (.0288) | -- |
|  | Age | **.38** (.0488) | -.03 (.578) |
|  | Sex | **.41** (.0316) | .07 (.849) |
|  | Education | **.49** (.0090) | .07 (.082) |
|  | VO_2_peak | **.45** (.0404) | -.01 (.831) |
| Language | -- | .23 (.233) | -- |
|  | Age | .22 (.281) | -.01 (.815) |
|  | Sex | .23 (.237) | .18 (.639) |
|  | Education | **.36** (.0460) | .07 (.087) |
|  | VO_2_peak | .25 (.279) | -.02 (.710) |
| Executive functions | -- | .32 (.098) | -- |
|  | Age | .25 (.193) | -.07 (.175) |
|  | Sex | .31 (.105) | -.08 (.826) |
|  | Education | .34 (.074) | **.09** (.0312) |
|  | VO_2_peak | .31 (.162) | .01 (.749) |
| Short-term memory | -- | .02 (.916) | -- |
|  | Age | -.06 (.751) | -.09 (.099) |
|  | Sex | .02 (.911) | .22 (.579) |
|  | Education | .10 (.647) | .04 (.355) |
|  | VO_2_peak | .02 (.939) | -.01 (.788) |
| Long-term memory | -- | .19 (.303) | -- |
|  | Age | .14 (.481) | -.06 (.225) |
|  | Sex | .19 (.311) | .06 (.868) |
|  | Education | .32 (.113) | .04 (.379) |
|  | VO_2_peak | .13 (.556) | .01 (.895) |
